# Development and Characterization of New Plant-Based Ice Cream Assortments Using Oleogels as Fat Source

**DOI:** 10.3390/gels10060397

**Published:** 2024-06-12

**Authors:** Sorina Ropciuc, Cristina Ghinea, Ana Leahu, Ancuta Elena Prisacaru, Mircea Adrian Oroian, Laura Carmen Apostol, Florina Dranca

**Affiliations:** Faculty of Food Engineering, Stefan cel Mare University of Suceava, 720229 Suceava, Romania; sorina.ropciuc@fia.usv.ro (S.R.); analeahu@fia.usv.ro (A.L.); ancuta.prisacaru@fia.usv.ro (A.E.P.); m.oroian@fia.usv.ro (M.A.O.); laura.apostol@fia.usv.ro (L.C.A.); florina.dranca@usm.ro (F.D.)

**Keywords:** candelilla wax, hemp seed oil, plant-based ice cream, millet milk, oat milk, oleogels, olive oil, physicochemical, rheological properties, spelt milk

## Abstract

The objective of this study was to develop candelilla wax oleogels with hemp seed oil and olive oil and use them as a fat source in the development of new plant-based ice cream assortments. Oleogels were structured with 3 and 9% candelilla wax and characterized by oil-binding capacity, peroxide value and color parameters. The oil-binding capacities of 9% wax oleogels were significantly higher than those of 3% wax oleogels, while peroxide values of oleogels decrease with increasing wax dosage. All oleogel samples are yellow-green due to the pigments present in the oils and candelilla wax. Physicochemical (pH, titratable acidity, soluble solids, fat, protein) and rheological (viscosity and viscoelastic modulus) parameters of plant-based ice cream mixes with oleogels were determined. Also, sensory attributes and texture parameters were investigated. The results showed that titratable acidity and fat content of plant-based ice cream samples increased with increasing wax percentage, while pH, soluble solids and protein values are more influenced by the type of plant milk used. The plant-based ice cream sample with spelt milk, hemp oil and 9% candelilla wax received the highest overall acceptability score. The hardness of the plant-based ice cream samples increased as the percentage of candelilla wax added increased.

## 1. Introduction

Nowadays, plant-based dairy alternatives have gained increasing popularity due to various factors such as: health issues (the number of people allergic to milk proteins and who are lactose intolerant has increased) [1], lifestyle changes (adoption of vegetarian and vegan diets) [2], environmental concerns (such as global pollution), animal welfare (related to human–animal interactions) [3] and religious beliefs [4]. According to Tachie et al. [5], plant-based dairy products account for 7.4% of the milk market and are expected to grow to 8.8% by 2031. In 2024, the non-dairy milk market in Europe was estimated at USD 5.60 billion [6]. Also, the consumption of plant-based milk in Europe increased by 14.1% in 2022 compared to 2021, with Germany being the leading market, followed by Spain [6]. The reasons for consuming alternative dairy products are: organic, high source of protein, low or no fat, lactose and gluten free, no additives, no added sugar, vitamin and mineral fortified and added calcium [7]. Milk sources of plant origin are: cereals (oats, spelt), pulses (soya, lupin), nuts (almonds, coconut), seeds (sesame, hemp) and pseudo-cereals (amaranth, quinoa) [5,8]. Cereal milks (oat, millet and spelt) were selected for the present study as raw materials for development of new frozen dessert assortments. Oat milk is a successful commercial cereal milk [9], millet milk is obtained from a gluten free cereal [10], while spelt milk has antistress properties and high nutritional content [11]. Oat (*Avena sativa* L.) is a cereal cultivated worldwide (Europe being the main producer in 2021) [12] and is rich in soluble fiber (oat β-glucan) that can have positive effects on human health (cholesterol lowering, reduction of postprandial glucose level, perception of satiety and stomach distention, reduction in cancer, immune-modulating properties) [13]. Oat milk (a water extract) is obtained from oatmeal [14] and is lower in protein and fat and higher in carbohydrates compared to cow’s milk [9]. Millets (*Poaceae grass* family), produced in many parts of the world (India being the largest millet producer in 2021) [15], are coarse cereals rich in carbohydrates (65–75%), dietary fibers (15–20%), antioxidants, vitamins and minerals and lower in protein (7–12%) and fats (2–5%) [16,17]. Millet milk contains carbohydrates (60–80%) and minerals and is gluten free, making it suitable for people with celiac disease or gluten intolerance and genetic disorders such as autism [18]. Spelt wheat (*Triticum aestivum* ssp. spelta) is an ancient crop (subspecies of common wheat) [19], cultivated in many European regions [20], which can grow in mountainous areas and on weak and less fertile soils [21]. This type of wheat has high contents of proteins, fats, antioxidants and phenolic acids [22]. Spelt milk contains protein (11–19%), starch (61–68%) [23], vitamins and minerals and can have an important satiating effect [24]. The proteins in spelt form gluten, which can cause symptoms in sensitive or allergic people [23]. Cereal milk is used as a raw material for the manufacture of various food products, such as: fermented oat milk products and beverages, products derived from oat milk (cheese, butter and powder), desserts (ice cream, cakes) [25], fermented foxtail millet beverages, milk-based pottage [26], millet ice cream [27,28], spelt-based ice cream, yogurt and cheese analogs [29]. Among the most popular plant-based food products is ice cream, a frozen product that preserves the properties of the ingredients used [30]. According to Teknoice [31], the plant-based ice cream market is expected to account for 10% of the total ice cream market by 2026. Although plant-based ice cream offers functional benefits, formulation of this product is more complicated and more ingredients are required to achieve the taste and textures demanded by consumers [32]. According to Airoldi et al. [33], fat has an important role in stabilizing the air phase and obtaining the sensory qualities of the product. Due to the lower fat content of plant milk, the addition of fat (in the form of oleogels, with more than 90% liquid vegetable oil [34]) must be considered when formulating plant-based ice cream [35]. Various vegetable oils (such as olive and hemp seed oils) can be used to develop oleogels [36]. Hemp (*Cannabis sativa* L.) seeds contain 30–35% oil (unsaturated fatty acids: 50–60% linoleic acid and 20–30% linolenic acid) [37], while 20% by weight of olives (*Olea europaea* L.) is oil content [38] (85% unsaturated acids: 70–83% oleic acid and linoleic or palmitoleic acid) [39]. Kozłowicz et al. [40] used hemp oil in the formulation of ice cream with (lactose-free) whole milk and investigated the potential of ultrasonic pasteurization to replace standard pasteurization. They observed a decrease in ice cream hardness with increasing hemp oil concentration, while the nutritional profile was improved due to the high phenolic content. Tagliamonte et al. [41] replaced cow’s milk cream with extra virgin olive oil in an ice cream formulation and observed that the ice cream did not significantly change its original flavor. Güven et al. [42] observed that the pH value of olive oil ice cream samples increased, and olive oil worsened ice cream melting. The properties of ice cream with oleogels from various seed oils (strawberry seed oil; tomato seed oil) were investigated by Nazarewicz et al. [43] and Nazarewicz et al. [44]. They observed that various characteristics of ice creams were affected with the increase in oleogel proportions.

The aim of this study was to investigate the possibility of reducing milk fat content in artisanal ice cream, replacing cream with hemp seed oil and olive oil using candelilla wax (3 and 9%) as a gelator. In addition, using plant-based milk (oat, millet and spelt) replacer can solve the problem of lactose absorption. Also, the research aims to determine the physiochemical (pH, titratable acidity, soluble solids, fat, protein) and rheological parameters of the obtained plant-based ice cream mix, as well as sensory attributes and texture parameters (hardness and adhesiveness) of plant-based ice cream samples.

## 2. Results and Discussion

### 2.1. Oleogel Characterization

**Figure 1:**
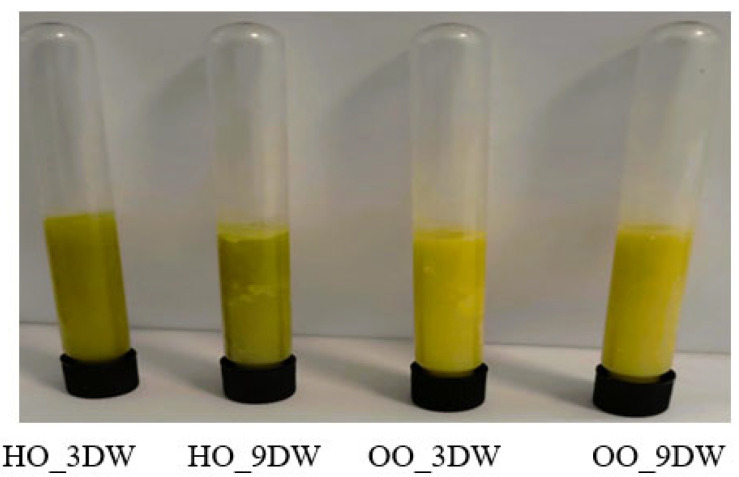
Oleogels formulated with candelilla wax and hemp and olive oils.

Oleogels obtained from hemp seed oil (HO) and olive oil (OO) with 3 and 9% candelilla wax (DW) are illustrated in Figure 1. The two wax concentrations of 3 and 9% were chosen in order to provide a physicochemical, textural and sensory comparison between a plant-based ice cream with a lower fat content and an ice cream with a medium fat content. The Romanian ice cream market is interested in lower fat products.

The functionality of oleogels in food products is ensured by the oil-binding capacity (interaction between liquid oil and oleogel molecules) of wax oleogels [45,46]. Waxes, such as candelilla wax (DW), have excellent gelling properties, oil-binding capacity (OBC) and easy availability and have been used in recent years in oleogel research [47]. OBCs of oleogels prepared with two different vegetable oils (HO and OO) and candelilla wax (3 and 9%) are presented in Table 1.

Higher OBC values (above 90%) were observed for oleogels with 9% DW, while OBC values below 75% were obtained for oleogel samples with 3% DW. According to Qu et al. [46], OBC values above 75% indicate that the samples were strongly gelatinized. OBC values are influenced by the percentage of DW, and Ghazani et al. [45] stated that oil loss can decrease when different proportions of wax are added to olive oil and a strong network structure is formed due to the high content of wax esters and hydrocarbons. Increasing the wax percentage will increase the OBC values [33,48]. The peroxide values (PVs) of the oleogel samples were determined 5 days after formulation of the oleogels and are presented in Table 1. According to Hamidioglu et al. [49], higher oxidative stability is indicated by lower peroxide values, and a product with PV between 1 and 5 meqO_2_/kg is classified as having a low oxidation state [50]. In the present study, the PVs of the oleogel samples are below 2 meq/kg, which means that all samples have a low oxidation state (are highly stable against oxidation) and were within the limit suggested by the Codex Alimentarius (15 meqO_2_/kg) [48]. The addition of candelilla wax can decrease the PVs of oleogel samples, as hemp oil can have PVs between 2.18 and 7.73 meqO_2_/kg on the first and sixth day of storage [49], while the PV for virgin olive oil during storage can vary between 11.6 and 15.3 meqO_2_/kg [51]. Peroxide values of oleogels decrease with increasing wax dosage (DW), leading to delayed oxidation reaction of unsaturated fatty acids present in oils. Color parameters of oleogel samples were determined and the results are presented in Table 1. L*, a* and b* values of oleogel samples are not statistically significantly different. The L* values ranged from 65.38 ± 13.2 (sample OO_9DW) to 71.49 ± 5.41 (sample HO_9DW), which means that the samples are lighter in color (L* = 100 white). Negative values were obtained for the color parameter a*, which means that all the samples tend towards green due to the color of the oils used (hemp and olive). All samples tend towards yellow (most likely influenced by the yellow color of the wax [52], or oil used) as positive values were recorded for the b* parameter. According to Núñez-García et al. [52], the color parameter values of the refined DW are L* = 62.06, a* = 10.53, b* = 37.95. Hemp oil is greenish in color due to chlorophyll pigments (L* = 18.13 ± 0.11, a* = 2.40 ± 0.36 and b* = 30.87 ± 0.14) [53]. Carotenes are responsible for the yellow color of olive oil, while chlorophylls contribute to the greenish color of some olive oils. According to Ayadi et al. [54], these pigments are also important for oil stability.

### 2.2. Physiochemical Analysis of Plant-Based Ice Cream Mix with Oleogels

**Figure 2:**
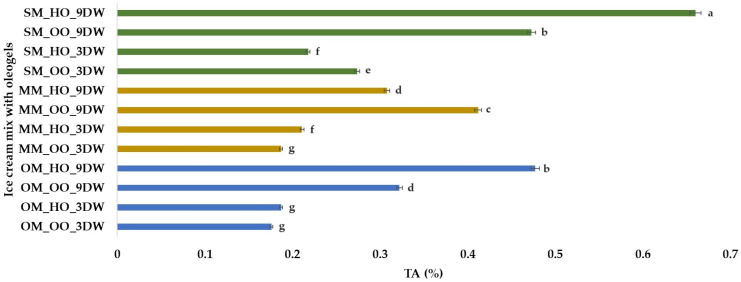
Titratable Acidity (%) of plant-based ice cream mix with oleogels. Means with different lowercase letters (a–g) indicate significant differences (*p* < 0.05) between samples.

Titratable acidity (TA, %) of plant ice cream mix with oleogels varied from 0.176% (OM_OO_3DW sample with oat milk (OM), oleogel from olive oil (OO) and 3% candelilla wax (3DW)) to 0.660% (SM_HO_9DW sample with spelt milk (SM), oleogel from hemp seed oil (HO) and 9% candelilla wax (9DW)) (Figure 2). Ozdemir [55] determined acidity between 0.12 and 0.18% for ice cream mixtures with oleogels, while Güven et al. [42] reported TA values of 0.17–0.19% for different vegetable oil ice creams. According to Masurovsky [56], the acidity of ice cream mix should be 0.25–0.30% to achieve the desired texture and flavor. The results of the present study showed that TA increased in the case of plant-based ice cream mix with spelt milk and oat milk (regardless of the type of oil used to obtain the oleogels) when the percentage of candelilla wax increased. Also, a greater increase in TA values was observed in the case of oleogels obtained with hemp oil compared to oleogels obtained with olive oil. The millet milk (MM) ice cream mix also had higher TA values for the samples with 9% candelilla wax, but a greater increase was observed for the MM_OO_9DW sample obtained with olive oil (Figure 2).

**Figure 3:**
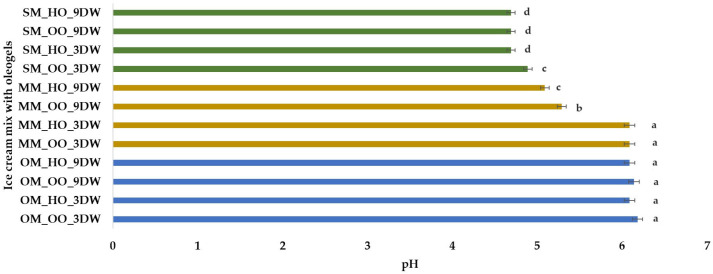
pH of plant-based ice cream mix with oleogels. Means with different lowercase letters (a–d) indicate significant differences (*p* < 0.05) between samples.

The pH of the plant ice cream mixture with oleogels ranges from 4.69 for sample SM_HO_3DW to 6.18 for sample OM_OO_3DW (Figure 3). These values are close to those reported by Ozdemir [55] (pH = 5.91–6.71) and Leahu et al. [30] (pH = 3.8–6.2). pH influences the perception of ice cream flavor and according to Homayouni et al. [57] should be about 6–7. The pH values for all oat milk ice cream mix samples do not show statistically significant differences (Figure 3), nor do the spelt milk ice cream mix samples (pH = 4.69), with the exception of sample SM_OO_3DW (pH = 4.89). In the case of the millet milk ice cream mix samples, it was observed that the pH values for the 3% DW samples were lower than for the 9% DW samples.

**Figure 4:**
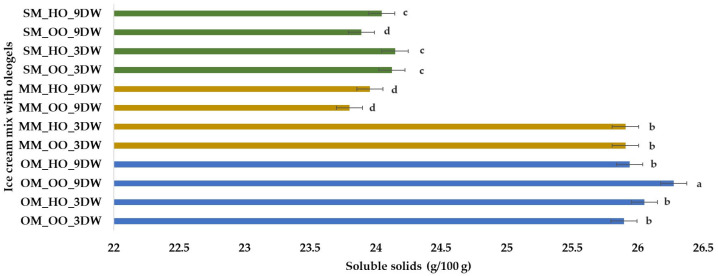
Soluble solids (g) per 100 g of plant-based ice cream mix with oleogels. Means with different lowercase letters (a–d) indicate significant differences (*p* < 0.05) between samples.

Results obtained for soluble solids (g) per 100 g of plant-based ice cream mix with oleogels are illustrated in Figure 4. It can be observed that the samples with oat milk had higher values, between 25.89 and 26.72 g soluble solids/100 g, followed by the samples with millet milk and 3% DW. The lowest values (about 23.88 g soluble solids/100 g) were obtained for samples MM_OO_9DW and SM_OO_9DW. The differences regarding the dry matter content for the analyzed mixes are explained by the fact that oats have a higher fat content (6–10%) than spelt wheat and most other cereals (2–3%). Oats have the highest fat content of all grains, with a high percentage of unsaturated fat. Another important compositional aspect is the protein content. The main storage proteins in oats are globulins (which are water soluble). Most cereals (including wheat and millet) rely heavily on prolamins as the main storage proteins, but oats are an exceptional case. Prolamins represent a minor percentage in oats. Current research on the composition of plant-based beverages has reported that the beverages are high in fat, carbohydrates and protein, which differ depending on the plant group from which they are derived. The soluble solids contents in samples formulated with oats, almonds and chestnuts were above 30 g/100 g, whereas, for samples formulated with coconut and soy, the values were below 30 g/100 g [58,59,60].

**Figure 5:**
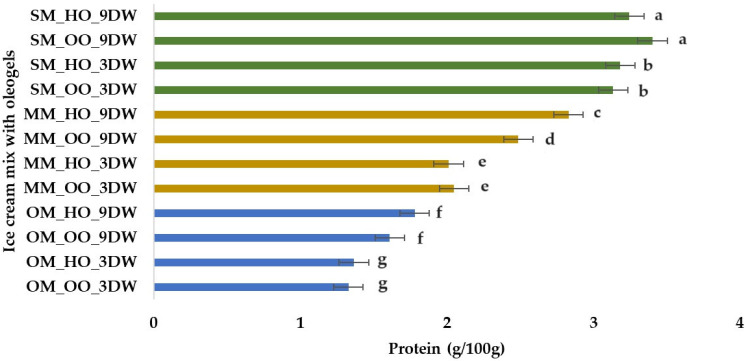
Protein (g) per 100 g of plant-based ice cream mix with oleogels. Means with different lowercase letters (a–g) indicate significant differences (*p* < 0.05) between samples.

Plant-based ice cream mix samples with spelt milk (3.13–3.40 g/100 g) had the highest protein content (Figure 5), followed by samples with millet milk (2.01–2.83 g/100 g) and oat milk (1.32–1.78 g/100 g). These values are consistent with those observed in other studies: 1.09–2.79% [30], 0.75–3.28% [61].

**Figure 6:**
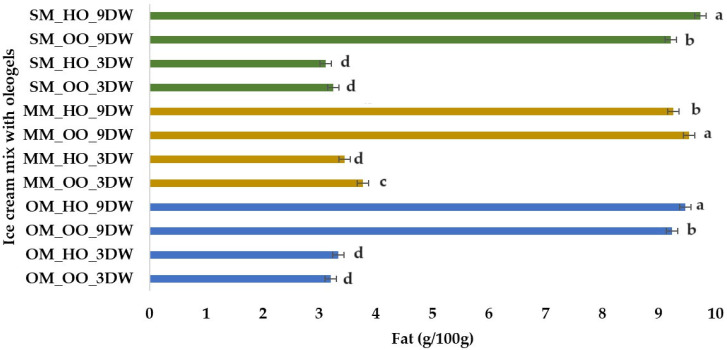
Fat (g) per 100 g of plant-based ice cream mix with oleogels. Means with different lowercase letters (a–d) indicate significant differences (*p* < 0.05) between samples.

Typically, the fat content of ice cream is around 8% and oleogels derived from vegetable oils are considered to be an alternative to fat [44]. Fat content (g) per 100 g of plant-based ice cream mix with oleogels is illustrated in Figure 6. The highest value was determined for the SM_HO_9DW sample (9.74 g/100 g), while the lowest value was observed for the SM_HO_3DW sample (3.12 g/100 g). All samples containing 9% DW have high fat content (over 9 g/100 g), regardless of the type of oil used to obtain the oleogels. Plant beverages are extracts of plant material dissolved in water, mimicking cow’s milk in consistency and appearance [62,63]. Homogenization and heat treatment are used to improve suspension and microbial stability of plant-derived beverages. The fat content in these drinks is low and varies depending on the vegetable source (cereals, legumes, nuts, seeds, pseudocereals). In the present research, sources of plants from the category of cereals and pseudocereals, with low fat content, were chosen. Among the types of vegetable drink used in the study, millet has the highest fat content (approximately 1.5–7%) and it differs a lot depending on the variety of millet (pearl, sorghum, finger, proso and foxtail millets). The fat content was increased by the oleogel incorporated in the mix mass used to obtain the frozen dessert. In the case of the drink based on spelt wheat with the lowest fat content, it can be explained by the fact that this sample formed the most stable emulsion, the oleogel being best incorporated into the product without separating into a mixing table.

### 2.3. Rheological Characterization of Plant-Based Ice Cream Mixes

**Figure 7:**
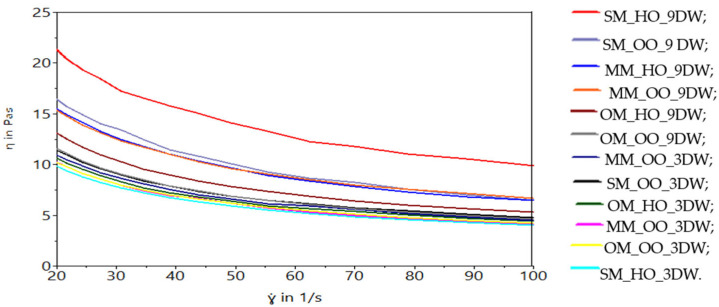
The viscosities of the plant-based ice cream mix.

The rheological behavior of the frozen dessert mix is much more complex than that of a simple liquid or, by comparison, that of a yogurt [64]. The mix matrix is a solution consisting of small (sugar) and large (polysaccharides, wax) molecules, in which particles from other phases (ice crystals, fat droplets and air bubbles) are suspended [64]. The effect of oleogel mixtures on the viscosity of the mixture at constant shear rate leads to obtaining curves with different values, related to the oleogel used. The viscosity curves of the plant-based ice cream emulsions are shown in Figure 7. The results show that the apparent viscosity for the frozen dessert mixes decreased with increasing shear rate over the entire shear rate tested between 20 s^−1^ and 100 s^−1^, reflecting non-Newtonian fluids with pseudoplastic behavior, indicating the formation of a weak droplet network structure [65]. The viscosity of the mix was determined at a temperature of 4 ℃ because the coagulation of the fat in the mix occurs at temperatures below this value and its role is to form the aerated structure, specific to the frozen dessert.

**Figure 8:**
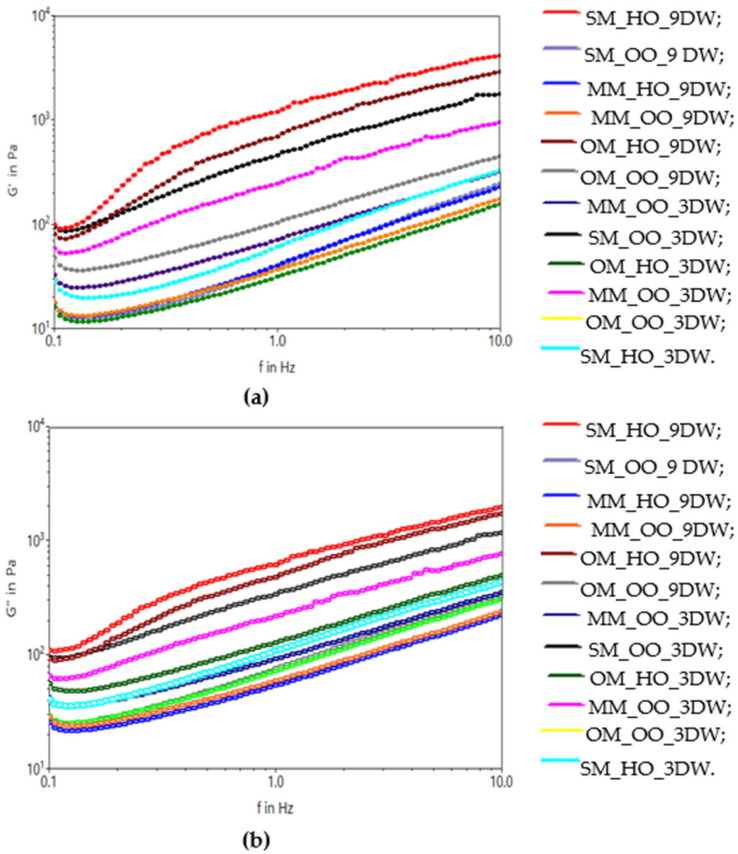
The viscoelastic moduli: (**a**) G′ and (**b**) G″ of the plant-based ice cream mix.

The elastic and viscous behaviors of the mixes are influenced by the addition of candelilla wax in the oleogel structure. There is a tendency to group the mixes formulated with 9% oleogel for the elastic modulus but also for the viscous modulus (Figure 8).

The mix formulated with spelt wheat drink (SM_HO_9DW) is differentiated, which has the best elastic and viscous properties. Frozen dessert mixes with a percentage of 9% showed significant reductions in viscosity with the increase in the amount of wax in the oleogel. In other words, the higher amount of candelilla wax in the product was responsible for the increased viscosity and reduced fluidity.

### 2.4. Sensory Evaluation of Plant-Based Ice Cream with Oleogels

**Figure 9:**
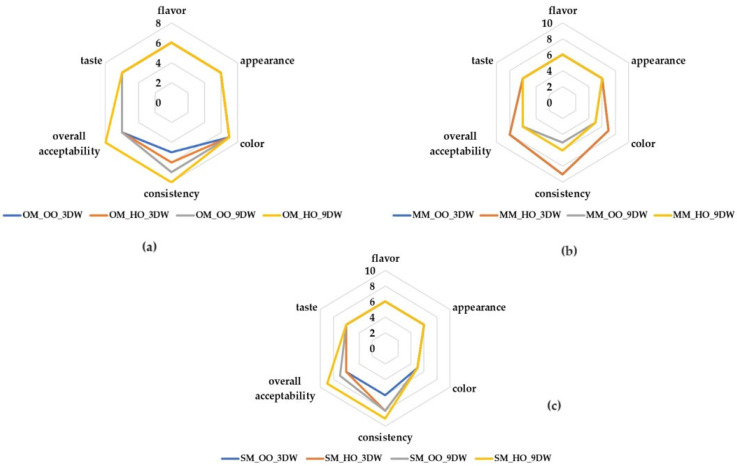
Sensory evaluation of plant ice cream from: (**a**) oat milk; (**b**) millet milk and (**c**) spelt milk with oleogels.

Figure 9 illustrates the sensory attributes for all plant-based ice cream samples. The sensory attributes of aroma, appearance and taste were equally valued, with no statistically significant differences between the scores. The color of all oat milk ice cream samples and millet milk ice cream with 3% DW was more appreciated than the other samples. Plant-based ice cream samples with oat milk and 9% DW were more appreciated for the consistency compared with the 3% DW samples (Figure 9a). Plant-based ice cream containing millet milk and 9% DW were more liked than samples with 3% DW in terms of consistency (Figure 9b). In the case of spelt milk ice cream, the highest score for consistency was received by sample SM_HO_9DW, followed by samples SM_OO_9DW and SM_HO_3DW (Figure 9c). The highest overall acceptability scores for plant-based ice cream with oleogels were achieved by the sample with spelt milk, hemp oil and 9% DW (SM_HO_9DW), followed by samples with oat milk, hemp oil and 9% DW (OM_HO_9DW); millet milk, hemp oil and 3% DW (MM_HO_3DW); and millet milk, olive oil and 3% DW (MM_OO_3DW).

### 2.5. Texture Analysis of Plant-Based Ice Cream with Oleogels

Texture parameters (hardness and adhesiveness) of plant ice cream with oleogels are presented in Table 2. Hardness values were higher for samples SM_HO_9DW, MM_HO_3DW, MM_OO_3DW and SM_OO_9DW than for the other samples. The lowest hardness value was recorded for sample MM_OO_9DW (516 ± 0.3). Akbari et al. [66] stated that ice cream hardness is influenced by fat content (low-fat ice cream will have a harder texture due to increased ice crystals) and also by overrun rate, ice crystal size and degree of fat instability [67].

The correlation between fat content and hardness of plant-based ice cream with oleogels was investigated using Pearson’s correlation and a negligible correlation (0.20) was observed between these two parameters. Plant-based ice cream hardness is affected by the addition of oleogels and the type of plant milk. It was observed that plant-based ice cream samples with oleogels from hemp seed oil and candelilla wax (9% DW) had higher hardness when spelt and oat milk were used. A use of a higher percentage of candelilla wax in oleogels leads to higher hardness [49,68]. However, in the case of plant-based ice cream with millet milk, oleogels from vegetable oils and candelilla wax (3% DW), high hardness values were recorded. Adhesiveness of plant-based ice cream samples decreased significantly (*p* < 0.05), while hardness increased.

### 2.6. Principal Component Analysis

**Figure 10:**
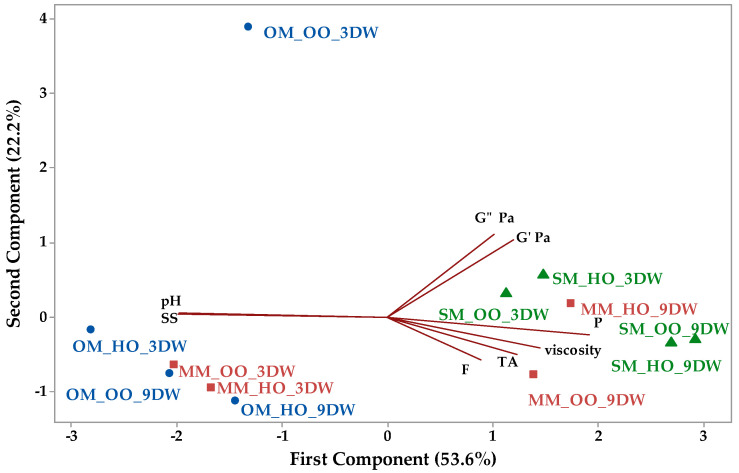
Principal component analysis biplot depicting the relationship between parameters of plant-based ice cream mix with oleogels (SS—soluble solids, F—fat, P—protein, TA—titratable acidity, G′—storage modulus and G″—loss modulus).

The relationship between physicochemical and rheological parameters of plant-based ice cream mix with oleogels is presented in Figure 10.

Principal component 1 (PC1) accounted for 53.6% of the total variation and had an eigenvalue of 4.29, while the second component (PC2) had an eigenvalue of 1.77 and accounted for 22.2% of the total variation. Most of the investigated parameters had positive loading on PC1, except pH and soluble solids which had negative loadings of −0.460 and −0.463, respectively. PC2 shows negative correlation with fat (−0.319), TA (−0.282), viscosity (−0.234) and protein (−0.134) and positive correlation with the other parameters. Figure 10 shows that all plant-based ice cream samples with spelt milk and oleogels were situated to the right in the score biplot and had positive values for PC1. Positive values for PC1 were also observed for the samples with millet milk and oleogels with candelilla wax (9% DW), while samples with millet milk and oleogels with candelilla wax (3% DW) had negative values for PC1. All plant-based ice cream samples with oat milk and oleogels were located on the left in score biplot and had negative values for both PC1 and PC2.

## 3. Conclusions

This study presents the possibility of using oleogels, obtained from candelilla wax, hemp seed oil and olive oil, in plant-based ice cream production. The properties of the plant-based ice cream are influenced by the type of plant milk and oleogels used. As the proportion of candelilla wax increased, the titratable acidity and fat content of plant-based ice cream samples increased. Plant-based ice cream samples with hemp seed oil oleogels and candelilla wax (9% DW) had higher hardness when spelt and oat milk were used and lower hardness when millet milk was used. Plant-based ice cream with 3% wax has a higher hardness and is crumbly, and the low fat content negatively influences aeration, but the type of vegetable milk used also influences its properties. Samples with 9% wax have more pleasant sensory and textural properties for consumers. This study showed that the addition of oleogel in the frozen dessert mix as a substitute for animal fat can be used to obtain a vegetable dessert similar to classic ice cream. This work also establishes that the addition of oleogels in the mix is advantageous for the general stability of the frozen dessert, and it increases the viscosity and elasticity which will lead to slightly aerated and creamy products. This method can also be applied to other frozen desserts based on fruit purees, fruit nectar and clear sweetened or natural fruit juices. Further investigation could be carried out on the magnitude of ice crystal development in fresh and stored soft serve by microstructural study. The textural properties of the frozen dessert and the sensory quality of the final product could be another aspect of the research, and it will also be essential to understand the optimal amount of oleogel required to obtain products with the pleasant flavor, smoothness and sensory properties appreciated by consumers.

## 4. Materials and Methods

### 4.1. Materials

Hemp seed oil (HO), olive oil (OO), oat milk (OM), millet milk (MM) and spelt milk (SM) were purchased from grocery stores (Suceava, Romania), while candelilla wax (DW) was supplied by Sigma-Aldrich (Hamburg, Germany). Sugar and flavors were purchased from a local supermarket.

### 4.2. Oleogel Preparation

The vegetable oils (HO and OO) used for the preparation of oleogels were obtained by cold pressing oilseeds. Candelilla wax is used as gelling agent in percentages of 3% and 9% (falling within the values mentioned in the literature from 0.5% *w*/*w* to 10.0% *w*/*w* [68]) and has the following characteristics: yellow color, melting point 68.0–72.0 °C, acid value 12.00–22.00, saponification value 45.00–65.00. It is obtained from the leaves of the candelilla shrub (*Euphorbia antisyphilitica*) and contains n-alkanes (C26–C33: tritriacontane, hentriacontane, triacontane, nonacosane and hexacosane), esters (C41: *m*/*z* 607; C39: *m*/*z* 579) and alcohols (penta-cyclic triterpenoids), sterols and free acids [69]. Oleogels were prepared according to the method described by Ropciuc et al. [48,68] and were stored at 4 °C until analysis. The coding of the oleogel samples was performed considering the type of vegetable oil and the percentage of wax added: HO_3DW (oleogel from hemp seed oil and 3% candelilla wax); OO_3DW (oleogel from olive oil and 3% candelilla wax); HO_9DW (oleogel from hemp seed oil and 9% candelilla wax); OO_9DW (oleogel from olive oil and 9% candelilla wax).

### 4.3. Preparation of Mixes for Plant-Based Ice Cream with Oleogels

Raw materials used to obtain the oleogel ice cream mix are: oat milk, millet milk and spelt milk, sugar, oleogel and flavors. The resulting mixture is homogenized in a blender and allowed to cool at 5 °C for 12 h. The mixture is placed in an Ice Cream Maker Model ICE-21R (Cuisinart, East Windsor, NJ, USA) and stirred for 20 min to form plant-based ice creams. After that, they were placed in a container and frozen at −17 °C for 48 h [70]. Table 3 presents the content of components used in plant-based ice cream mix formulation.

### 4.4. Preparation of Plant-Based Ice Cream with Oleogels

Frozen plant-based ice cream mixes were prepared in 300 mL batches with a base formula of 10% fat, 10% banana, 5% sucrose, 0.5% xanthan gum, 0.5% vanillin and 75% plant-based beverage (Table 4). The 10% fat comes from the addition of oleogel formulated from different varieties of vegetable oil and percentages of wax of 3 and 9%. The frozen dessert is produced in four different stages: mixing the ingredients, pasteurization, homogenization and aeration and freezing. The mixing process is designed to mix the vegetable drink with banana, xanthan gum and sugar. The mixture is mixed until it becomes homogeneous and pasteurized at 65–70 °C for 30 min. After pasteurization, it is cooled and poured into a homogenizer (Klarstein vanilly sky family) which is designed to freeze and incorporate air into the structure at −18℃. Freezing is performed at −20 °C after hardening the mix in the homogenizer.

### 4.5. Oil-Binding Capacity (OBC)

According to Ropciuc et al. [48], 1 g of each oleogel sample was placed in Eppendorf tubes and stored at 4 °C for 1 h, after which they were centrifuged in a microcentrifuge (Hermle Z206A) for 15 min at 4000 rpm and 24 °C, then the liquid fraction was removed. Oil-binding capacity was calculated based on the equation provided by Ropciuc et al. [48].

### 4.6. Determination of the Peroxide Index (PV) of Oleogels

The standard methodology ISO 3960:2007 [71] was used for determination of the peroxide index of oleogels. PV was calculated based on the equation provided by Ropciuc et al. [48].

### 4.7. Color Measurement of Oleogels

The CIELab system (Konica-Minolta 200, Tokyo, Japan) was considered for the color measurement of oleogels. L* (lightness/darkness), a* (redness/greenness), b* (yellowness/blueness) parameters were determined.

### 4.8. Physiochemical Analysis of Plant-Based Ice Cream Mix with Oleogels

pH of plant-based ice cream mixtures with oleogels was determined using a pH meter (Fisher, Waltham, MA, USA) according to the AOAC 14.022 method [72], titratable acidity (TA) was determined by titration of samples with 0.1 N NaOH in the presence of phenolphthalein as an indicator considering the AOAC 920.124 method [73].

Total solids of plant-based ice cream mixtures with oleogels were measured according to the gravimetric method [74]. Fat and protein contents were determined according to Leahu et al. [30].

### 4.9. Rheological Characterization of Plant-Based Ice Cream Mix with Oleogels

Rheological characteristics of plant-based ice cream mix with oleogels were investigated using a dynamic rheometer (Haake Mars 40, Karlsruhe, Germany). Viscosity and viscoelastic modulus were determined according to Leahu et al. [30] and Ropciuc et al. [68].

The plate/plate geometry (40/80 mm) was used at a gap of 2 mm and a temperature during work of 4 °C. The rheometer was set in a rotational mode with shear rates increasing from 1 to 100 s^−1^. After pouring the mix onto the plate a waiting time of 300 s was applied to reach equilibrium before measurement. The mixture was measured in triplicate at 4 °C. Flow curves were obtained by measuring viscosity as a function of shear rate from 20–100 s^−1^. Viscoelastic properties were determined using the frequency module at variable intensity in the interval 0.1–100 Hz.

### 4.10. Sensory Analysis of Plant-Based Ice Cream with Oleogels

Flavor, appearance, color, consistency, taste and overall acceptability of plant-based ice cream with oleogels were evaluated by a panel of 21 members selected from the students of the Faculty of Food Engineering. Sensory evaluation was performed according to the ISO 8587:2006 standard and a 9-point hedonic scale (from 1 = “Dislike Extremely” to 9 = “Like Extremely”, with 5 = “Neither Like nor Dislike”) was considered [30].

The evaluators analyzed the frozen desserts from the point of view of taste, smell, appearance, color and general acceptability. The sensory analysis was carried out by the evaluators after signing the agreement “I am aware that my answers are confidential and I agree to participate in this sensory evaluation”. The sensory evaluation criteria of the frozen dessert are described in Table 5.

### 4.11. Texture Analysis of Plant-Based Ice Cream with Oleogels

Texture attributes of hardness and cohesiveness were obtained by texture profile analysis. For the compression tests in two cycles, a TVT-6700 texturometer (Perten Instruments, Hägersten, Sweden) was used with an aluminum cylinder of 25 mm in diameter. To produce force–time curves, frozen dessert samples (ø, 40 mm; h, 60 mm) were fixed in the center of the platform during the test using a sterile glass container (Petri dish). The tests were performed as follows: a pre-test speed of 5.0 mm s^−1^, a post-test speed of 2.0 mm s ^−1^, compression strain 45%. The experiments were performed at room temperature (23 ± 1 °C). The texture analysis was performed for the freshly frozen dessert in the homogenizer (Klarstein vanilly sky family). Hardness (N) was recorded as the maximum force during the first compression cycle. Cohesiveness was considered as the ratio of the positive force area under the second to the first compression cycle. Three replicates were performed.

### 4.12. Statistical Analysis

Statistical evaluation was performed with Minitab version 17 (Minitab, Inc., State College, PA, USA). An analysis of variance (ANOVA) with a 95% confidence interval (*p* < 0.05) and Tukey’s test were considered to compare the results obtained. Principal component analysis was also performed.

## Figures and Tables

**Table 1:** Results of oil-binding capacity (OBC), peroxide value (PV) and color parameters of oleogels.

Sample	OBC (%)	PV (meqO_2_/kg)	L*	a*	b*
HO_3DW	73.36 ± 4.65 ^b^	1.837 ± 1.24 ^a^	67.56 ± 4.19 ^a^	−6.818 ± 0.54 ^a^	23.92 ± 6.47 ^a^
HO_9DW	95.59 ± 4.27 ^a^	1.259 ± 0.35 ^a^	71.49 ± 5.41 ^a^	−8.624 ± 0.77 ^a^	25.65 ± 7.42 ^a^
OO_3DW	61.72 ± 6.36 ^b^	1.853 ± 0.59 ^a^	69.42 ± 5.28 ^a^	−7.901 ± 1.03 ^a^	30.71 ± 3.01 ^a^
OO_9DW	91.80 ± 6.43 ^a^	0.732 ± 0.61 ^a^	65.38 ± 13.2 ^a^	−7.677 ± 1.27 ^a^	26.50 ± 9.50 ^a^

Different superscript letters in the same column indicate significant difference between values at *p* < 0.05 level.

**Table 2:** Hardness and adhesiveness of plant-based ice cream with oleogels.

Sample	Hardness (g)	Adhesiveness (g.s)
OM_OO_3DW	547 ± 0.10 ^j^	−24 ± 0.02 ^b^
OM_HO_3DW	642 ± 0.30 ^g^	−37 ± 0.01 ^c^
OM_OO_9DW	762 ± 0.10 ^f^	−68 ± 0.02 ^f^
OM_HO_9DW	951 ± 0.20 ^e^	−81 ± 0.05 ^h^
MM_OO_3DW	1181 ± 0.10 ^c^	−90 ± 0.02 ^j^
MM_HO_3DW	1351 ± 0.06 ^b^	−127 ± 0.00 ^l^
MM_OO_9DW	516 ± 0.3 ^k^	−19 ± 0.02 ^a^
MM_HO_9DW	547 ± 0.50 ^j^	−48 ± 0.07 ^d^
SM_OO_3DW	559 ± 0.05 ^i^	−56 ± 0.00 ^e^
SM_HO_3DW	562 ± 0.40 ^h^	−74 ± 0.01 ^g^
SM_OO_9DW	1081 ± 0.61 ^d^	−80 ± 0.00 ^i^
SM_HO_9DW	1652 ± 0.20 ^a^	−112 ± 0.01 ^k^

Different superscript letters in the same column indicate significant difference between values at *p* < 0.05 level.

**Table 3:** The content of components used in plant-based ice cream mix formulation.

Sample Coding	Main Sample Ingredients
SM_HO_3DW	Spelt milk (SM), oleogel from hemp seed oil (HO) and 3% candelilla wax (3DW)
SM_HO_9DW	Spelt milk (SM), oleogel from hemp seed oil (HO) and 9% candelilla wax (9DW)
SM_OO_3DW	Spelt milk (SM), oleogel from olive oil (OO) and 3% candelilla wax (3DW)
SM_OO_9DW	Spelt milk (SM), oleogel from olive oil (OO) and 9% candelilla wax (9DW)
MM_HO_3DW	Millet milk (MM), oleogel from hemp seed oil (HO) and 3% candelilla wax (3DW)
MM_HO_9DW	Millet milk (MM), oleogel from hemp seed oil (HO) and 9% candelilla wax (9DW)
MM_OO_3DW	Millet milk (MM), oleogel from olive oil (OO) and 3% candelilla wax (3DW)
MM_OO_9DW	Millet milk (MM), oleogel from olive oil (OO) and 9% candelilla wax (9DW)
OM_HO_3DW	Oat milk (OM), oleogel from hemp seed oil (HO) and 3% candelilla wax (3DW)
OM_HO_9DW	Oat milk (OM), oleogel from hemp seed oil (HO) and 9% candelilla wax (9DW)
OM_OO_3DW	Oat milk (OM), oleogel from olive oil (OO) and 3% candelilla wax (3DW)
OM_OO_9DW	Oat milk (OM), oleogel from olive oil (OO) and 9% candelilla wax (9DW)

**Table 4:** The plant-based ice cream preparation.

Ingredient	Quantity per 100 g
Millet/spelt wheat/oat vegetable drink	75 mL
Oleogel	10 g
Banana	10 g
Sugar	4 g
Xanthan gum	0.5 g
Flavor: vanillin	0.5 g

**Table 5:** Sensory evaluation criteria of the plant-based ice cream.

Index	Evaluation Standards	Score
Color	Uniform color, milky white	8–9
	Almost milky white	6–7
	Matte and tends to be gray	4–5
	Unacceptable	1–3
Aroma	No oleogel/oil flavor	8–9
	Low oleogel/oil flavor	6–7
	Perceptible oleogel/oil aroma	4–5
	Strong oleogel/oil aroma	1–3
Taste	Moderate sweetness, pleasant taste	8–9
	Moderate sweetness, slightly oily	6–7
	Plain or slightly oily taste	4–5
	Unpleasant or oily taste	1–3
Aspect	Uniform system, with aerated structure	8–9
	Relatively uneven system, slightly aerated	6–7
	Non-uniform, non-aerated system	4–5
	Unstable, unventilated system	1–3

## Data Availability

The original contributions presented in the study are included in the article, further inquiries can be directed to the corresponding author.
